# Contamination of a Water Stream and Water Drainage Reaching Matosinhos Beach by Antibiotic-Resistant Bacteria

**DOI:** 10.3390/microorganisms11122833

**Published:** 2023-11-22

**Authors:** Matilde A. Pereira, Josman D. Palmeira, Helena Ferreira

**Affiliations:** 1 Laboratory of Microbiology, Department of Biological Sciences, Faculty of Pharmacy, University of Porto, 4050-313 Porto, Portugal; mat.az.pereira@gmail.com (M.A.P.); josmandantasp@gmail.com (J.D.P.); 2 i4Health, UCIBIO, University of Porto, 4050-313 Porto, Portugal

**Keywords:** antibiotic-resistant bacteria, carbapenemases, fecal contamination, Matosinhos beach, One Health, Portugal, recreational water

## Abstract

Antibiotic-resistant bacteria represent a major public health concern, especially impacting medical care centers and hospitals, thereby challenging the effectiveness of current infection treatment protocols. The emergence and persistence of antimicrobial resistance in the environment have been thoroughly researched, with a focus on the aquatic environment as a potential reservoir of these bacteria in areas with anthropogenic contamination. Having this in mind, this work aims to investigate the water streams of Riguinha and Brito Capelo Street, both of which ultimately flow into Matosinhos Beach in Portugal, to determine the potential presence of fecal contamination. Six water samples were collected and analyzed within twenty-four hours from these two water streams. A phenotypic characterization was performed in various volumes on MacConkey agar with antibiotics. Randomly selected lactose-fermenting gram-negative bacteria underwent antimicrobial susceptibility tests using the agar diffusion method following EUCAST guidelines, covering β-lactam and non-β-lactam antibiotics. The isolates were analyzed through Polymerase Chain Reaction. The findings of this study confirm that both water streams were contaminated by multidrug-resistant bacteria such as Enterobacteriaceae, including *Escherichia coli*, the KESC group, and *Pseudomonas,* exhibiting extended-spectrum β-lactamases (ESBL), AmpC β-lactamases, and carbapenemases. These indicate the presence of fecal contamination with relevant antimicrobial-resistant threats.

## 1. Introduction

Multidrug-resistant Enterobacteriaceae poses a global public health threat due to limited treatment options, which undermine the effectiveness of the current range of antibiotics [1]. Enterobacteriaceae (including *Escherichia coli*, *Klebsiella* spp., and *Enterobacter* spp.) are gram-negative bacteria which inhabit a mammal’s intestinal tract, as well as soil, water, sewage, and food. They are among the most usual human opportunistic pathogens, being responsible for community and healthcare-related diseases like gastroenteritis, pneumonia, urinary tract infections, and meningitis. As antibiotic resistance can also be detected in animals and in the environment, the One Health concept has been implemented by the World Health Organization, emphasizing interconnected health systems (WHO) [2]. These bacteria have acquired resistance to the antibiotics most used and prescribed to treat infections, such as β-lactams (penicillin, cephalosporins, and carbapenems), over the years [3].

The misuse of antibiotics significantly contributes to the emergence of antibiotic resistance. On the one hand, over-consumption of antibiotics not only kills beneficial bacteria in our system, our natural and indispensable microbiota, but also enhances the growth of both antibiotic-resistant commensal and pathogenic microorganisms. On the other hand, the underuse of antibiotics, wherein individuals fail to complete the prescribed treatment, provides an opportunity for resistance to develop. It is imperative to use antibiotics wisely and responsibly to ensure their effectiveness now and future [4].

In terms of resistance mechanisms, extended-spectrum β-lactamases (ESBL) producing Enterobacteriaceae (E-ESBL) are relevant opportunistic pathogens considered a threat to human health. They are responsible for treatment failures in severe infections and silent colonization of the community population’s intestinal tracts, with a particularly notable impact on the elderly. The presence of E-ESBL is not confined to hospital environments; they can be found in several ecological niches, such as in the environment. ESBLs are enzymes responsible for the hydrolysis of oxyimino-β-lactam antibiotics, considered significant therapeutic agents for treating severe human and animal infections. CTX-M, SHV, and TEM are the most prevalent types of these enzymes globally, but numerous other variants exist [5]. Among CTX-M enzymes, CTX-M-1, CTX-M-14, and CTX-M-15 are the most widespread in human-related settings and are frequently associated with E-ESBL reports. This suggests that anthropogenic pressure upon natural environments has a strong influence on antimicrobial resistance emergence, including the dissemination of genes encoding these enzymes. The CTX-M-15 variant is responsible for infectious outbreaks worldwide and is often linked to high-risk clones of *Escherichia coli*, such as CTX-M-15/ST131 *E. coli.* This clone is accountable for extraintestinal infections resistant to antibiotics. The increasing frequency of infections caused by *E. coli* isolates producing ESBLs is a serious concern, as the efficacy of third generation cephalosporins against this pathogen is progressively diminishing [3].

AmpC β-lactamases are widely distributed and can also inactivate β-lactam antibiotics such as cephalosporins. Initially believed to be chromosomal, they have been recognized as plasmid-mediated cephalosporinases as well, since the 1990s. These enzymes contribute to therapeutic failures in community-acquired infections, especially involving *E. coli*, *Klebsiella pneumoniae*, and *Salmonella enterica*. The most disseminated AmpC cephalosporinase globally is CMY-2, associated with *Enterobacterales* infections in humans [3]. AmpC β-lactamases cannot degrade carbapenems; however, they block their action by binding to them. Plasmid-encoded AmpC CMY-2 is often found in Enterobacteriaceae like *E. coli*, causing global carbapenem resistance [6].

Carbapenems were considered the antibiotics of choice for treating infections caused by extended-spectrum β-lactamase and AmpC producers [1]. Carbapenem-resistant Enterobacteriaceae (CRE) are currently increasing globally, posing a major threat as carbapenems are last-line antibiotics, and leading to longer hospital stays, increased costs, and mortality. The World Health Organization (WHO) categorizes CRE as critical drug-resistant bacteria, and the Centers for Disease Control (CDC) identifies *Klebsiella* spp., *Escherichia coli*, and *Enterobacter* spp. as prominent emerging concerns in carbapenem resistance [1]. CRE are not only nosocomial and community-acquired pathogens but are also found in food-producing animals and in the environment. Key resistance mechanisms include carbapenemases production (β-lactamase enzymes), altered or mutated porins, enhanced efflux pump-action, and modified penicillin-binding proteins. Carbapenemases, hydrolytic enzymes, come in non-metallo and metallo forms, catalyzing carbapenem hydrolysis using serine or zinc as active catalytic substrates. The genes that encode theses enzymes are often located on plasmids or associated with mobile elements (transposons or integrons), facilitating their spread [7]. Carbapenemases mainly belong to three β-lactamase classes: Ambler A (*Klebsiella pneumoniae* carbapenemase, e.g., bla_KPC_), B or metallo-β-lactamases (e.g., bla_IMP_, bla_VIM_, bla_NDM_), and D or oxacillinases (e.g., bla_OXA-48_), which are carried either on chromosomes or acquired via plasmids [8]. KPC- and NDM-producing bacteria have shown resistance to most β-lactams, fluoroquinolones, and aminoglycosides. As healthcare facilities can function as reservoirs for CRE, KPC-producing *Klebsiella pneumoniae* has already been associated with hospital outbreaks across Europe. OXA-48-like carbapenemases, when associated with extended-spectrum β-lactamases, can also create high resistance levels [7]. Since carbapenems are not entirely metabolized within the body, some residues found in human excreta may enter hospital sewage. This raises the concern of pathogens in hospital effluent developing resistance to carbapenems due to the selective pressure upon them. It is thought that hospital sewage may act as a reservoir for resistance genes, allowing organisms to acquire resistance through horizontal gene transfer events. Likewise, antibiotic residues discharged into wastewater along with human feces could contribute to the selection of carbapenemases-producing Enterobacteriaceae in water sources [7]. Resistance evolves continuously in the Enterobacteriaceae family [1].

A standardized international terminology was established to harmonize definitions for various patterns of bacterial resistance. This initiative not only improved the comparability of surveillance data but also facilitated global, regional, and local epidemiological assessments of public health impact. Multidrug-resistant (MDR) is characterized by non-susceptibility to at least one agent in three or more antimicrobial categories. Extensively drug-resistant (XDR) is defined by non-susceptibility to at least one agent in all but two or fewer antimicrobial categories (bacterial isolates susceptible to only one or two categories). Lastly, Pandrug-resistant (PDR) means non-susceptibility to all agents in all antimicrobial categories [9].

Contamination of environmental water, including bathing water, through the release of sewage, fecal discharge, agricultural runoff, and healthcare effluents containing huge amounts of antimicrobial-resistant organisms could seriously impact water quality and jeopardize the health of vulnerable people [4,10]. It is imperative that significant measures be taken to reduce infection rates, especially those requiring antibiotic treatment, by identifying environments contaminated with antibiotic-resistant bacteria [11,12].

**Figure 1:**
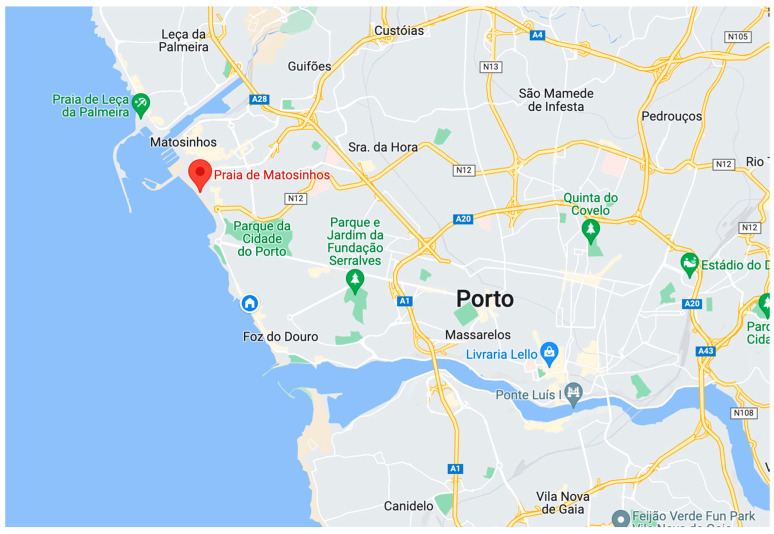
Matosinhos beach, Matosinhos, Portugal.

Matosinhos Beach is a highly frequented beach in Portugal (Figure 1), drawing visitors not only during the peak summer season but also throughout the winter months. This enduring popularity can be attributed to the presence of numerous surf schools. However, recent concerns have arisen regarding the water quality at this beach, prompting several inspection initiatives and water analysis. Reports have shed light on health issues experienced by surfers who frequent Matosinhos Beach, including gastrointestinal discomfort and skin irritations, which may be related to elevated levels of bacteria, notably intestinal *Enterococci* and *Escherichia coli*, detected in the waters [13]. Visitors to this beach are often discouraged from swimming due to bacteriological contamination, and it is suspected that one contributing factor could be the streams that flow into the beach [14,15].

**Figure 2:**
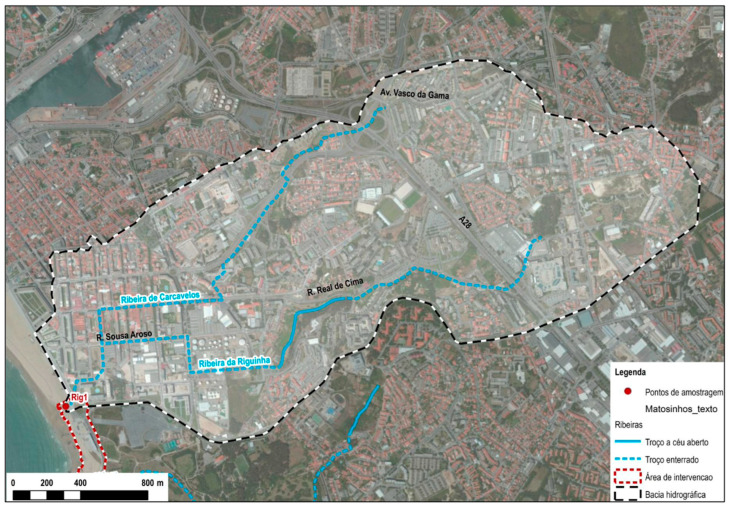
Route map of Riguinha water stream [16].

The rainwater drainage system of Riguinha is mainly situated within the municipality of Matosinhos, though it also receives contributions from the municipality of Porto, encompassing the region spanning from Praça da Cidade de Salvador to Edifício Transparente. This drainage network is bifurcated into two main branches, corresponding to the hydrographic sub-basins of the Riguinha and Carcavelos streams (as illustrated in Figure 2) [16].

The Riguinha stream originates at Fonte das Sete Bicas and runs approximately 3.8 km before merging with the boundary between Matosinhos Beach and International Beach. Meanwhile, the Carcavelos stream, originating near Feira da Senhora da Hora, runs 2.7 km before converging with the Riguinha stream, near the intersection of Sousa Aroso Avenue and D. João I Street (Figure 2) [16]. As both water streams are in a highly urbanized and densely populated area, only a small section of the Riguinha stream is in the open, next to the Parque Real, in Matosinhos. In 2014, water analyses already revealed elevated concentrations of *E. coli,* suggesting the presence of fecal contamination in this stream. Bathing water quality is one of the most important criteria for the beach to earn a blue flag designation and often requires structural changes to domestic wastewater and rainwater drainage networks [16].

This present study aims to investigate two water streams: the Riguinha natural water stream and the water drainage from Brito Capelo Street, both of which flow into Matosinhos beach. The objective is to assess possible fecal contamination and antimicrobial resistance in selected isolates. For this purpose, several water samples were analyzed, and the authors performed a phenotypic and genotypic characterization, including Polymerase Chain Reaction (PCR) detection of resistance genes for the most relevant bacteria identified.

## 2. Materials and Methods

### 2.1. Water Sampling

This research involved the collection of six water samples. Three of these samples were obtained from the Riguinha stream, while the remaining three were taken from Brito Capelo Street’s drainage. To minimize the risk of contamination, all water samples were collected using gloves into a sterile Schott container with a capacity of 1 L, which was opened only at the moment of collection. All samples were collected between October 2021 and April 2022. The first one was on 20 October at 3:35 p.m., the second on 29 November at 7:15 p.m., the third and fourth on 10 February at 10:40 a.m. and 11:25 a.m., respectively, and finally the fifth and sixth were on 11 April at 2:30 p.m.

**Scheme 1:**
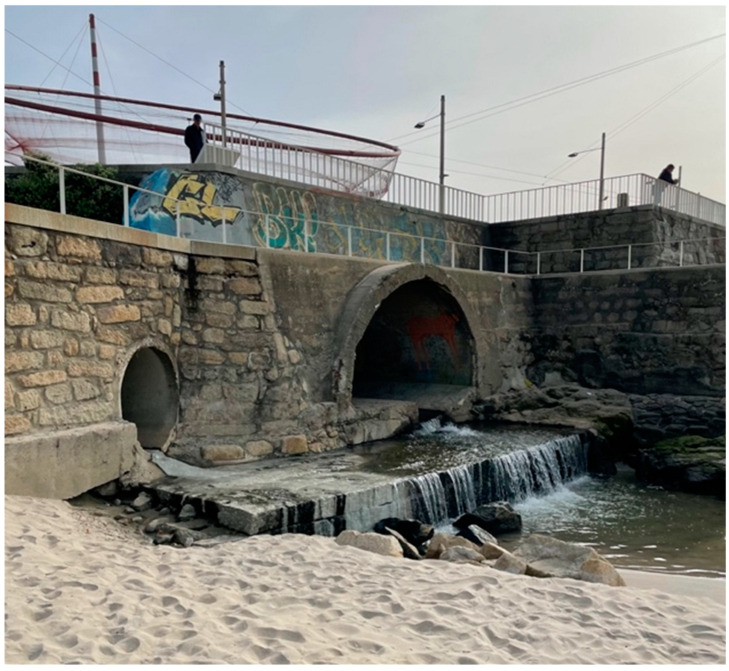
Tunnel on the right: final section of Riguinha stream, concrete pipe 4.4 m wide and 2.3 m high. On the left: smaller concrete pipe corresponding to the water drainage of Brito Capelo Street, Matosinhos.

**Scheme 2:**
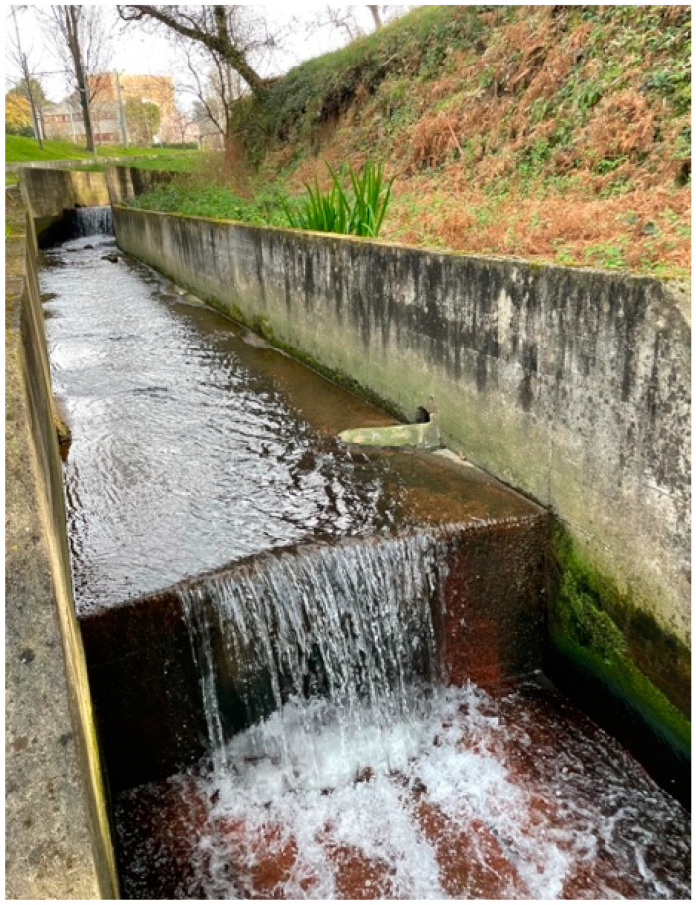
Open-air section of Riguinha stream near Parque Real, Matosinhos.

The samples were coded based on their collection location as follows: the first two samples (MAT1 and MAT2) were obtained from the water drainage system on Brito Capelo Street; the third and fourth (MAT3 and MAT4) from Riguinha stream—specifically, from the mouth of the stream and the open access section, respectively—and the fifth (MAT5) once again from the mouth of Riguinha stream. The last sample (MAT6) was collected from Brito Capelo Street drainage once more. For visual reference, Scheme 1 and Scheme 2 illustrate the precise locations from which theses samples were acquired. All the flow captured by the rainwater drainage system of Riguinha stream and Brito Capelo Street flows into Matosinhos Beach (Scheme 1).

### 2.2. Culture Medium Preparation

For this study, MacConkey agar, MacConkey agar with antibiotics (ampicillin, cefotaxime, and meropenem), and Mueller–Hinton mediums were used, both sourced from Liofilchem, Italy. MacConkey agar is a selective medium for gram-negative bacteria and differential, whereas Mueller–Hinton is a non-selective and non-differential medium. MacConkey agar medium was prepared following the manufacturer’s instructions. In cases where antibiotics were required, they were added to the medium before distribution, as antibiotics can be degraded by high temperatures. The volume of antibiotic added to each medium was calculated, considering the final concentration needed, the initial concentration of the stock solution, and the final medium volume (400 mL). The final concentrations were 100 μg/mL for ampicillin, 1 μg/mL for cefotaxime, and 0.5 μg/mL for meropenem. After solidifying, the medium was ready for use. The same procedure was followed for preparing the Mueller–Hinton medium.

Finally, all the mediums were tested using *E. coli* ATCC 25922 (ATCC—American Type Culture Collection, Manassas, VA, USA), as a control method. For the mediums to be considered valid, bacterial growth was expected in MacConkey agar but should have been absent in MacConkey agar with antibiotics, as this strain lacks acquired resistance to these antibiotics.

### 2.3. Water Filtration

**Scheme 3:**
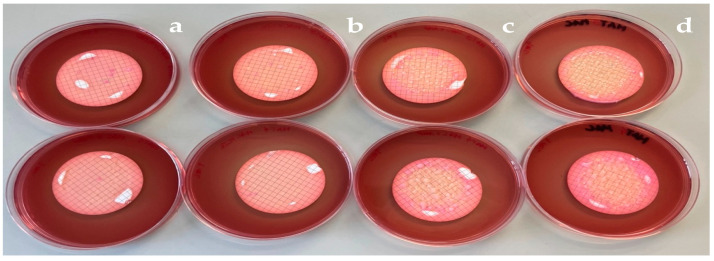
Membranes of 1 mL filtration after 24 h incubation at 37 °C placed in MacConkey agar (**d**), MacConkey agar with meropenem (**a**), with cefotaxime (**b**), and ampicillin (**c**).

To initiate the research, the standard method of membrane filtration was performed on all water samples within 24 h of collection [17]. A sterile cellulose acetate membrane with a pore size of 0.2 μm and a diameter of 47 mm, sourced from the Advantec MFS™ brand (Dublin, CA, USA), was used. This specific pore size allowed the capture of bacteria on top of the membrane, as the bacteria are smaller than the pore size. In the initial exploratory approach, sample volumes of 100 mL and 10 mL were used. However, the bacterial growth was so dense, with no isolated colonies, that it was necessary to modify the approach. As a result, subsequently tested volumes were of 1 mL (with a 1/20 dilution using sterile water) and 100 μL, by spread using a sterile glass Pasteur pipette in L format. Isolated bacterial colonies were obtained only from the last two volumes. The membranes from each filtration volume were placed on both MacConkey agar and MacConkey agar supplemented with ampicillin, cefotaxime, and meropenem. They were then incubated overnight at 37 °C. Scheme 3 depicts the membranes from the 1 mL filtration placed on MacConkey agar, both with and without antibiotics. A noticeable difference in bacterial growth density was observed between MacConkey agar without antibiotics (Scheme 3d) and MacConkey agar with meropenem (Scheme 3a).

### 2.4. Phenotypic Characterization

The colonies obtained from the filtration membranes were spread in new culture media to achieve isolated and pure colony-forming units (cfu). Mainly lactose-fermenting, gram-negative bacteria were chosen, at random, from MacConkey agar with cefotaxime and meropenem for further phenotypic characterization, as these bacterial isolates are more relevant. Some lactose non-fermenting bacteria were also analyzed, such as *Pseudomonas* spp., making a total of 38 isolates (16 from Brito Capelo Street and 22 from Riguinha stream).

Antimicrobial susceptibility tests were conducted using the Mueller–Hinton medium for all target isolates, employing the agar diffusion method according to EUCAST [18,19]. This included the assessment of β-lactams, non-β-lactams, and specific antibiotics for *Pseudomonas* [19].

The placement of antibiotic disks, sourced from Liofilchem, Italy, or OXOID, United Kingdom, was performed with a specific aim to detect synergisms between oxyimino β-lactam antibiotics and the combination of amoxicillin with clavulanic acid that was centrally positioned on the plates. Each disk was applied with a consistent relative distance of 20 mm between them.

The antibiotics used, along with their abbreviations and disk contents (μg), were as follows:

- β-lactam antibiotics: amoxicillin-clavulanic acid (AMC/AUG30), ampicillin (AMP10), aztreonam (ATM30), cefepime (FEP30), cefotaxime (CTX30), cefoxitin (FOX30), ceftaroline (CPT30), ceftazidime (CAZ30), and meropenem (MRP/MEM10).
- Non β-lactam antibiotics: chloramphenicol (C30), ciprofloxacin (CIP5), fosfomycin (FO200), gentamicin (GEN/CN10), nitrofurantoin (F300), tigecycline (TGC15), tetracycline (TE30), tobramycin (TOB10), and trimethoprim-sulfamethoxazole (SXT25).
- Specific antibiotics for *Pseudomonas*: doripenem (DOR10), aztreonam (ATM30), cefepime (FEP30), levofloxacin (LEV5), ceftazidime (CAZ10), gentamicin (GEN10), meropenem (MRP10), tobramycin (TOB10), netilmicin (NET30), amikacin (AK30), ceftazidime (CAZ30), piperacillin + tazobactam (TZP110), piperacillin (PRL100), and ciprofloxacin (CIP5).

Additionally, a quality control assessment of the antibiogram disks was conducted using a suspension of *E. coli* ATCC 25922. This was performed to ensure that inhibition zones fell within acceptable ranges. Results were compared to EUCAST quality control (QC) tables [20]. The inhibition zone diameter for each antibiotic was measured with a digital caliper in millimeters and compared with the EUSCAST Breakpoint Tables to categorize the isolates as standard dosing regimen (S), susceptible, increased exposure (I), or resistant (R) [21].

Bacterial isolates were presumptively identified based on their color on Chromogenic UTI Medium from Himedia, Maharashtra, India, which allows the identification of bacteria typically associated with urinary tract infections. Bacteria from the KESC group (*Klebsiella* spp., *Enterobacter* spp., *Serratia* spp., and *Citrobacter* spp.) exhibited blue-colored colonies, *Pseudomonas* displayed yellow colonies, and *Escherichia coli* showed pink/purple colonies.

An oxidase test was performed to confirm that some isolates were *Pseudomonas*, while the S.I.M test was used to distinguish between *E. coli* and the KESC group. The oxidase strips used were from Liofilchem, Roseto degli Abruzzi, Italy, and the S.I.M medium was from Microbact, Durham, UK. The oxidase test was employed to determine the presence of the cytochrome c oxidase. This test is useful for differentiating between the families *Pseudomonadaceae* (oxidase-positive) and Enterobacteriaceae (oxidase-negative). The S.I.M test was used to evaluate whether the isolates could produce hydrogen sulfide, their motility, and their ability to produce indole from tryptophan, with the addition of Ehrlich reagent to the medium.

Regarding the resistance mechanisms, AmpC producers were confirmed via phenotypic tests with inhibitors (cloxacillin), ESBL producers using double-disk synergy test, while carbapenemase producers were detected using Carbapenem Inactivation Method (CIM). To identify AmpC producers, we measured the difference in inhibition zones between cefoxitin (FOX30) and cefoxitin with cloxacillin (FOC230). An isolate was classified as an AmpC producer if the difference was greater than or equal to 3.5 mm. Furthermore, these isolates had to exhibit resistance to CTX, CAZ, AMC, and FOX, while being susceptible to FEP, following the guidelines outlined in EUCAST [22].

ESBL producers were confirmed via the characteristic synergism observed when the antibiotic disks cefotaxime (CTX30) and amoxicillin-clavulanic acid (AMC30) were brought together (20 mm), or/and if the difference between the inhibition zones for cefotaxime (CTX30) and cefotaxime + clavulanic acid (CTL40) was greater than or equal to 3.5. mm. Additionally, the isolates had to exhibit resistance to CTX and CAZ, while remaining susceptible to FOX and AMC [22].

**Scheme 4:**
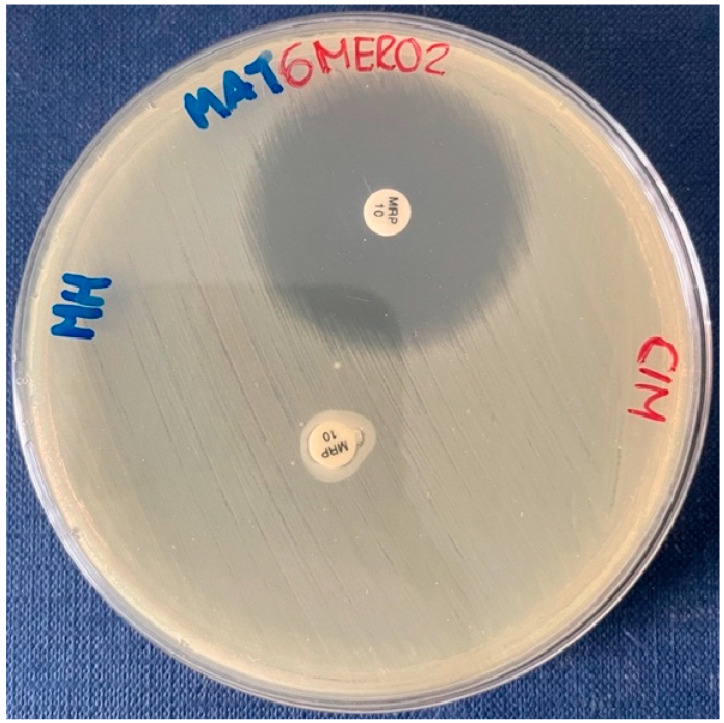
CIM positive for MAT6MERO2 isolate, as the meropenem in the susceptibility test disk was inactivated, allowing *E. coli* ATCC25922 to grow. On the contrary, the antibiotic on the control disk inhibited the growth of this strain.

Lastly, carbapenemase producers were identified via the Carbapenem Inactivation Method (CIM), a phenotypic test that detects carbapenemase activity in gram-negative rods within eight hours. It can reliably detect activity encoded by various β-lactamase genes such as *bla*_KPC_, *bla*_NDM_, *bla*_OXA-48_, *bla*_VIM_, *bla*_IMP_, and *bla*_OXA-23_ in species of Enterobacteriaceae (e.g., *Klebsiella pneumoniae* and *Escherichia coli*), but also in non-fermenters (e.g., *Pseudomonas aeruginosa*). In order to perform the CIM, a 10 μL inoculation loop of culture, taken from a Mueller–Hinton, was suspended in 400 μL water on an Eppendorf tube. Subsequently, a susceptibility-testing disk containing 10 μg meropenem was immersed in the suspension and incubated for a minimum of two hours at 37 °C. Afterwards, the disk was removed from the suspension using an inoculation loop, placed on a Mueller–Hinton agar plate inoculated with a susceptible *E. coli* indicator strain (ATCC 29522), and then incubated overnight at 37 °C. This indicator strain inoculation was performed with a suspension of 0.5 McFarland, spread in Mueller–Hinton agar using a sterile cotton swab. A positive result meant that the bacterial isolate produced carbapenemase, and the meropenem in the susceptibility disk was inactivated, allowing uninhibited growth of the susceptible *E. coli* ATCC25922, as demonstrated in Scheme 4. A negative one meant that the bacterial isolate did not produce carbapenemases and a clear inhibition zone was observed [23].

### 2.5. Genotypic Detection

The genotypic characterization was only performed on bacterial isolates from samples MAT5 and MAT6 (14 isolates), as representative samples of the bacteria present in the water of Riguinha stream and the water drainage from Brito Capelo Street, respectively. DNA extraction was carried out from all the bacteria of interest using the alkaline lysis method. The purpose of this procedure was to isolate DNA for further analysis.

Procedure:

1. 20 mL of alkaline lysis buffer, consisting of 0.05 M NaOH and 0.25% sodium dodecyl sulfate (SDS), was added to a 0.2 mL Eppendorf tube to suspend 2 to 3 bacterial colonies in the solution.
2. The bacterial suspension was then incubated in a thermal cycler at 99 °C for 15 min. During this incubation, the detergent (SDS) disrupted cell membranes, allowing the alkaline environment to denature both chromosomal and plasmid DNA.
3. Following incubation, 180 mL of Tris-EDTA (TE 1x) buffer solution was gently added, and the solution was homogenized before centrifuging at 12,000 rpm for 15 min.
4. The resulting supernatant, approximately 150 mL, was carefully removed and transferred to a new Eppendorf tube for storage. With the addition of a neutralization buffer, the DNA was renatured and a white precipitate that consisted of SDS, lipids, and proteins was formed.

The extracted DNA was stored at 4 °C for a few months (up to six months), but it could also be kept at −20 °C for long-term preservation if necessary. MAT5 and MAT6 isolates were screened via Polymerase Chain Reaction. A multiplex PCR for detection of the most frequent acquired carbapenemase genes, including *bla*_NDM_, *bla*_OXA-48_, and *bla*_KPC_, was performed. All CIM-positive bacterial isolates, as well as those exhibiting resistance to meropenem or reduced susceptibility to this antibiotic, were tested.

In this process, 2 μL of total DNA were subjected to multiplex PCR in a 25 μL reaction mixture. The mix for detecting the mentioned genes included SuperHot Master Mix Taq Polymerase, from BIORON (concentration 2x), along with each primer (forward and reverse) and sterile ultra-pure water. For positive controls, we used standard controls obtained from DNA extraction of well-characterized strains carrying these resistance genes. One control was of *bla*_KPC_ and the other of *bla*_OXA_ (OXA-48, TEM, SHV). For negative control, water was used instead of DNA.

Amplification was carried out with the following thermal cycling conditions: 10 min at 94 °C (bacteria lysis and release of DNA); 36 cycles of amplification consisting of 30s at 94 °C (denaturation), 40 s at 52 °C (primer-specific annealing), and 50 s at 72 °C with 5 min at 72 °C for the final extension [24]. Table 1 shows the primers and their sequences (5′–3′) of sense primer (F) and antisense primer (R) that were used in this PCR to amplify specific fragments of the resistance genes and the product size (bp).

A second Multiplex PCR for rapid detection of genes encoding CTX-M extended-spectrum β-lactamases was performed. This assay allowed the authors to detect and distinguish alleles-encoding CTX-M enzymes from all five phylogenetic groups. Specific primers for alleles-encoding enzymes belonging to groups 1, 2, 8, 9, and 25 were used. The tested *bla*_CTX-M_ genes groups, primer pairs, and predicted amplicon sizes are presented in Table 2. Fragments of alleles-encoding enzymes of groups 8 (666 bp) and 25 (327 bp) were amplified using two specific forward primers and a shared reverse primer. Once more, 2 μL of total DNA were subjected to multiplex PCR in a 25 μL reaction mixture. The only variation in the detection mix was the set of primers used [25].

Only two positive controls were applied: +24 (B0HH CTX-M-15 (G1)) and +25 (KP18 SHV-2, CTX-M-2 (G2)). Amplification was carried out with the following conditions: initial denaturation at 94 °C for 5 min; 30 cycles of 94 °C for 25 s, 52 °C for 40 s, and 72 °C for 50 s, and a final elongation step at 72 °C for 6 min [25].

Lastly, two Simplex PCR assays were performed to detect *bla*_AmpC_ genes, in this specific case: *bla*_dha-1_, *bla*_dha-2_ and *bla*_CMY_. For this PCR, the genes tested, primer pairs (forward and reverse), and predicted amplicon sizes are presented in Table 3. For the detection of *bla*_dha-1_ and *bla*_dha-2_ genes, the amplification conditions were as follows: 3 min at 94 °C; 25 amplification cycles consisting of 30 s at 94 °C, annealing at 64 °C 30 s, and 1 min at 72 °C, with 7 min at 72 °C for the final extension [26]. For the detection of *bla*_CMY_ gene, amplification conditions were as follows: 5 min at 94 °C; 35 amplification cycles consisting of 1 min at 94 °C, 1 min at 49 °C, and 1 min at 72 °C, with 7 min at 72 °C for the final extension [27].

PCR products were identified through agarose gel electrophoresis, incorporating Sybr^®^ Safe as DNA intercalating dye. The results were observed with a transilluminator and saved using a BIO-RAD Gel DocTM XR+ System, with Image LabTM Software (https://www.bio-rad.com/en-cn/product/image-lab-software?ID=KRE6P5E8Z accessed on 28 September 2023). The agarose gel was prepared by adding 1–2% agarose (GeneOn) in sterile water and heating in the microwave until a clean solution was obtained, followed by the addiction of 3.5 mL of GreenSafe Premium (nzytech). The amplicons’ sizes were compared to a DNA ladder (NZYDNA Ladder VI from nzytech). A loading Buffer II (from BIORON (concentration 6x)) was mixed with every PCR product before initiating this procedure.

For the separation of Multiplex PCR products, carbapenemases and extended-spectrum β-lactamases DNA fragments were analyzed via electrophoresis in a 2% agarose gel at 90 V for 90 min in 1xTAE (40 mmol/L Tris–HCl [pH 8.3], 2 mmol/L acetate, 1 mmol/L EDTA) containing 0.05 mg/L ethidium bromide [24]. On the other hand, for separation of the Simplex PCR products, AmpC DNA fragments were analyzed via 1.5% agarose gel electrophoresis at 120 V with a running time of 30 min in 1XTAE.

## 3. Results

### 3.1. Water Filtration

A surprisingly abundant bacterial growth was observed on all membranes of the various filtration volumes performed on each collected water sample. These samples were collected randomly, considering different time frames and climate conditions. Bacteria from three distinct groups (*Pseudomonas*, KESC, and *E. coli*) were consistently obtained. The observation of isolated colony-forming units was achieved by either spreading 100 µL on MacConkey agar with meropenem or cefotaxime, or by using 1 mL filtration membranes placed on MacConkey agar with meropenem.

**Scheme 5:**
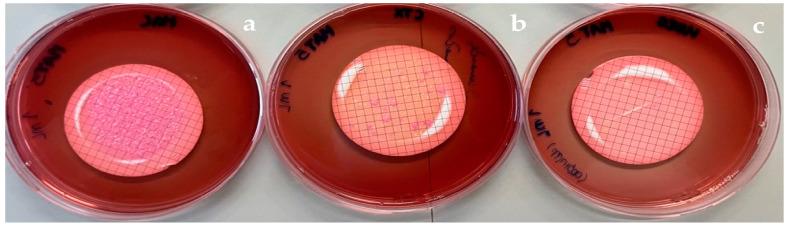
1 mL filtration membranes of MAT5 water sample placed in MacConkey agar (**a**), with cefotaxime (**b**), and with meropenem (**c**).

The reduction in bacterial growth became quite evident when comparing larger and smaller volumes of filtration membranes (100 mL–1 mL with dilution and 100 µL). Similar trends were observed when comparing bacterial growth on MacConkey agar and MacConkey agar with antibiotics, particularly meropenem, as illustrated in Scheme 5. While the authors conducted a random selection of lactose-fermenting isolates resistant to CTX and MRP, it is important to note that all isolates observed were analyzed. Thus, our interpretation of the results obtained was primarily qualitative. Lactose-fermenting and non-fermenting bacteria were found, the latter in large numbers, especially in the MAT6 sample.

### 3.2. Bacterial Identification

Among all the bacteria found in the water samples, a total of 38 bacterial isolates were analyzed. Most of these isolates were lactose-fermenting bacteria, which were our main interest; nevertheless, some non-fermenting bacteria were also studied (based on their relevance in terms of resistance). From the water drainage of Brito de Capelo Street (MAT1, MAT2 and MAT6 samples), we analyzed 16 isolates, and from the Riguinha water stream (MAT3 and MAT5 samples from the mouth and MAT4 from the open access section) came 22 isolates.

At first analysis via Chromagar Orientation medium, we presumptively identified five *Escherichia coli*, six KESC, four *Pseudomonas,* and one isolate that did not belong to any of these three groups, from the Brito Capelo Street samples. In the case of the Riguinha stream samples, we identified fifteen *Escherichia coli*, seven KESC, and three *Pseudomonas*. Two of the *Pseudomonas* identified were presumptively *Pseudomonas aeruginosa*, due to their green coloration in the UTI medium.

### 3.3. Antimicrobial Resistance Phenotype

All the results regarding the inhibition zone diameters obtained for each tested antibiotic, as well as the categorization of the isolates as susceptible (S—green cell), susceptible with increased exposure (I—white cell), or resistant (R—red cell), are presented in Table 4. From the results of the antibiograms, we concluded that the majority of the isolates exhibited resistance to β-lactam antibiotics, including amoxicillin-clavulanic acid (AMC) to fifth-generation cephalosporins such as ceftaroline (CPT). Two isolates out of 38 in total were resistant to all β-lactams antibiotics tested (*E. coli*—MAT5MERO1 and KESC group—MAT6MERO2).

We also observed resistance and reduced susceptibility to carbapenems, particularly meropenem (MRP), in a significant number of isolates. Those classified as “susceptible with increased exposure (I)” already possess carbapenemases and tested positive in the CIM test. In contrast, fewer isolates showed resistance to gentamicin (8 isolates), nitrofurantoin (5 isolates), trimethoprim/sulfamethoxazole (11 isolates), and chloramphenicol (7 isolates). They were 26 out of 38 multidrug-resistant isolates.

Regarding the antibiograms performed with antibiotics specific for *Pseudomonas*, we measured the inhibition zone diameters for each antibiotic and compared them with the EUCAST Guidelines. However, our primary focus was on Enterobacteriaceae isolates, particularly those resistant to carbapenems due to their clinical significance. In terms of the resistance mechanisms, we identified Enterobacteriaceae bacteria that produce ESBLs, AmpC enzymes, and carbapenemases (Table 4). Carbapenemase-producing isolates exhibited resistance to meropenem or reduced susceptibility, although a few isolates with inhibition zone diameters still considered susceptible tested positive in the CIM test.

Regarding the prevalence of these three different resistance mechanisms in the two water samples from the Riguinha stream and Brito Capelo Street, we found that in the first water sample, there were 9 ESBL-producing isolates, 8 carbapenemase-producing isolates, and 2 AmpC-producing isolates out of a total of 22 isolates. In the second sample, we identified 7 AmpC producers, 4 carbapenemase producers, and 2 ESBL producers out of 16 isolates.

In conclusion, it is essential to highlight our findings related to bacterial contamination in the water samples collected at different points along the Riguinha stream and the water drainage system of Brito Capelo Street. Upstream of Riguinha (MAT4), our analysis predominantly revealed the presence of ESBL-producing bacteria. However, downstream at MAT3 and MAT5, we identified carbapenemase-producing bacteria.

### 3.4. Genotypic Characterization

**Figure 3:**
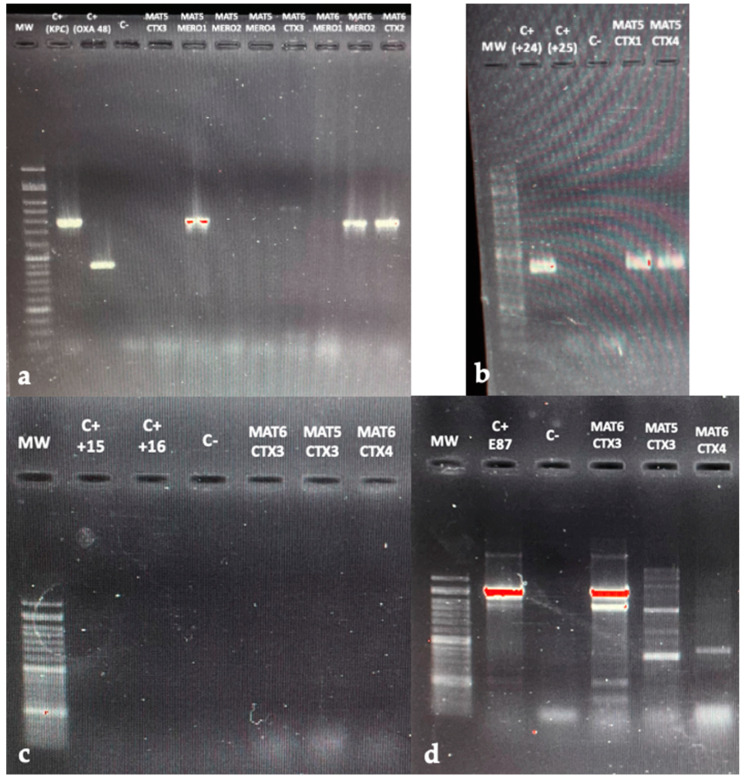
(**a**) Agarose gel electrophoresis (2%) used for separation of the different multiplex PCR products, performed to detect carbapenemase genes. (**b**) Multiplex PCR assay for *bla*_CTX-M_ alleles from G1, G2, G8, G9, and G25. (**c**) Agarose gel electrophoresis (1.5%) used for separation of the different multiplex PCR products performed to identify AmpC *bla*_DHA_ gene. (**d**) Agarose gel electrophoresis (1.5%) used for separation of the different multiplex PCR products performed to identify AmpC *bla*_CMY_ gene. Lanes labeled “MW”—correspond to DNA ladder; “C+”—to positive controls used, and “C−“—blank control.

A 2% agarose gel electrophoresis was performed to separate the products of the Multiplex PCR used for detecting acquired carbapenemase genes, as presented in Figure 3a. Positive controls for bla_KPC_ and bla_OXA-48_ yielded the expected bands, confirming the specificity of the primers used.

Numerous isolates from the last two water samples were tested, including MAT5CTX3, MAT5MERO1, MAT5MERO2, MAT5MERO4, MAT6CTX3, MAT6MERO1, MAT6MERO2, and MAT6CTX2. All of these isolates were found to be carbapenemase-producers, as confirmed by the CIM test.

Specifically, MAT5MERO1, previously identified as *E. coli*, MAT6MERO2, and MAT6CTX2, both identified as KESC, carried the *bla*_KPC_ gene, as evidenced by the amplification of the corresponding gene, resulting in a 798 bp band. In contrast, all other isolates, while confirmed as carbapenemase-producers, tested negative for the *bla*_KPC_, *bla*_OXA-48_, and *bla*_NDM_ genes, which are among the most frequently identified carbapenemases genes. Testing for other potential resistance genes, such as *bla*_VIM_ and *bla*_IMP_, was the next step; however, it was not possible due to time constraints. Additionally, there was no band observed in the negative control lane, indicating that gene amplification was not affected by PCR products.

The results of the Multiplex PCR assay for detecting genes-encoding CTX-M extended-spectrum β-lactamases, specifically from groups 1, 2, 8, 9, and 25, are presented in Figure 3b. We conducted tests on two isolates: MAT5CTX1, identified as *E. coli*, and MAT5CTX4, identified as KESC. These isolates had previously been classified as ESBL-positive. Upon analyzing the agarose gel electrophoresis data, we concluded that these isolates carried the *bla*_CTX-M-group1_ gene. This conclusion was based on the presence of a 415 bp band, which matched the positive control 24.

Finally, the results of the two simplex PCR assays performed to detect bla_AmpC_ genes, namely bla_DHA_ and bla_CMY_, are presented in Figure 3c,d, respectively. The AmpC-positive isolates tested included: MAT6CTX3 (KESC), MAT5CTX3 (*E. coli*), and MAT6CTX4 (KESC). In the first electrophoresis (Figure 3c), no bands appeared in the positive controls used, as they did not contain the bla_DHA_ gene responsible for the phenotype, and no suitable controls were available in the laboratory. All three isolates tested negative for this gene.

Regarding the second electrophoresis, it was difficult to interpret, since nonspecific bands appeared, which was not expected. It appears to have amplified the positive control’s *bla*_CMY_ gene correctly, and there was no interference by the PCR reaction products, as the negative control did not display any bands. However, the presence of multiple bands in the isolates introduced uncertainty. To draw definitive conclusions, we will need to repeat the PCR. There is a possibility that the MAT6CTX3 isolate carries the *bla*_CMY_ gene, since it displayed a band similar to that of the positive control (Figure 3d). Still, we cannot confirm this with absolute certainty.

## 4. Discussion

In these watercourses we found ESBL −, AmpC −, and carbapenemase-producing Enterobacteriaceae, as well as *Pseudomonas*, exhibiting multidrug resistance. It is important to note that these bacteria are supposedly of environmental origin, found in two water streams that flow into the beach, which was unexpected. Also, there is a possibility that bacteria may acquire resistance mechanisms through the aquatic environment, which could lead to their spread and subsequent further transmission [10]. Some of these bacteria demonstrated reduced susceptibility and resistance to meropenem. The decreased susceptibility is also concerning, as bacteria classified as “susceptible with increased exposure (I)” already possess carbapenemases, which confer some level of resistance.

Carbapenem resistance rates rose alarmingly in Portugal, especially among *Klebsiella pneumoniae*, between 2014 and 2017. In several healthcare settings in the north of Portugal, the ST147 *K. pneumoniae* clone presenting K-type K64 has been one of those responsible for KPC-3 spread. Also, it was responsible for the first large outbreak of carbapenemase-producing Enterobacteriaceae in a hospital in the north of Portugal in 2015. Beyond that, clones, including *E. coli* ST131 and carbapenemases, predominantly KPC-3 but also OXA-48 and VIM, were identified three years after the onset of carbapenemases spreading in this hospital [28].

In contrast, fewer isolates showed resistance to gentamicin (CN), nitrofurantoin (F), trimethoprim/sulfamethoxazole (SXT), and chloramphenicol, indicating that these antibiotics are still effective against infections caused by these bacteria. Applying the definitions for MDR (multidrug-resistant), XDR (extensively drug-resistant), and PDR (pandrug-resistant) as defined by Magiorakos [9], we identified several MDR bacteria in the water samples. For example, MAT3MERO6, a KESC isolate, exhibited resistance to meropenem, tetracycline, and tobramycin.

In addition, we highlight the fact that higher contamination levels were observed at the mouth of the river. This discrepancy suggests a potential contamination source from the open section of the Riguinha stream, possibly due to accidental wastewater discharge or contamination from human and animal feces. Unfortunately, we were unable to pinpoint the exact source and location of contamination. Further research is required to address this critical knowledge gap.

Similarly, the water drainage system of Brito Capelo Street may also be susceptible to accidental contamination. It should be emphasized that rainwater from such systems is typically discharged into the environment without treatment or control measures. This situation poses a significant risk to public health and calls for immediate attention and action. Two isolates belonged to the KESC group, and one *E. coli* carried the *bla_KPC_* gene. This finding aligns with the prevalence and widespread distribution of the KPC carbapenemase globally. Despite its initial discovery in *Klebsiella pneumoniae* isolates, clinical isolates of KPC-producing *Escherichia coli* and certain species of *Klebsiella* spp. and *Enterobacter* spp. have also been identified [6].

In conclusion, relevant acquired genes commonly found in clinical isolates such as *bla*_KPC_, and particularly in Enterobacteriaceae, were detected in bacteria from these water samples. A similar study on the occurrence of CRE in a Portuguese river also identified multidrug-resistant isolates, including *K. pneumoniae* carrying the KPC carbapenemase-encoding gene, specifically *bla*_KPC-3_ [29].

Regarding the MAT5CTX1 isolate, it is likely to contain CTX-M-15, as *E. coli* strains carrying CTX-Ms are known to be widespread ESBL-producing bacteria, with CTX-M-15 being the most commonly detected resistance gene. However, within the scope of our testing conditions, we can only confirm the presence of the *bla*_CTX-M-group1_ gene [30]. In a related investigation focusing on wastewater and river water in Tunisia, ESBL-producing *Escherichia coli* and *Klebsiella pneumoniae* were also isolated. Most of these isolates exhibited multidrug resistance and were found to produce CTX-M-15 [31].

In the context of future research, it will be imperative to expand the scope by analyzing a more extensive range of water samples collected from both streams and during different seasons throughout the year. This broader analysis aims to comprehensively understand the impact on water quality. Furthermore, analyzing a larger number of bacterial isolates and assessing their resistance, along with identifying the responsible genes, will provide a more accurate characterization of contamination levels and associated threats.

## 5. Conclusions

The findings of this study allowed us to confirm the presence of antibiotic-resistant bacterial contamination in both the Riguinha stream and the rainwater drainage system of Brito Capelo Street in Matosinhos. Surprisingly, from very small volumes of water samples, we were able to detect great contamination with multidrug-resistant bacteria, including ESBL-producing, AmpC-producing, and carbapenemase-producing Enterobacteriaceae. It is highly likely that these waters have been contaminated by wastewater or fecal discharges. However, we were unable to pinpoint for certain the source and exact location where the contamination occurred. Therefore, there is a need for further research in this area. The results of this study also highlight the importance of assessing water quality, as environmental water can act as reservoirs for these bacteria, enabling their dissemination and posing a significant threat to public health. Consequently, the information retrieved from this investigation could be used to inform bathing water management and policy in Matosinhos beach. Finally, looking to the future, carbapenem resistance in Enterobacteriaceae is expected to continue to increase substantially, limiting more and more the therapeutic options and jeopardizing public health. Hence, it is critical to control water quality and stop the spread of these bacteria.

## Figures and Tables

**Table 1:** Genes that confer resistance to carbapenems that were tested, nucleotide sequence (5′–3′) of sense (F) and antisense (R) primers used, and PCR product size for each primer (bp).

Genes	Primers Sequences (5′–3′)	Amplicon Size (bp)
***bla*_OXA-48_**	**OXA-F** GCGTGGTTAAGGATGAACAC	438
	**OXA-R** CATCAAGTTCAACCCAACCG	
***bla*_NDM_**	**NDM-F** GGTTTGGCGATCTGGTTTTC	621
	**NDM-R** CGGAATGGCTCATCACGATC	
***bla*_KPC_**	**KPC-Fm** CGTCTAGTTCTGCTGTCTTG	798
	**KPC-Rm** CTTGTCATCCTTGTTAGGCG	

**Table 2:** Groups of CTX-M genes that were tested, nucleotide sequence (5′–3′) of sense (F) and antisense (R) primers used, and PCR product size for each primer (bp).

Genes	Primers Sequences (5′–3′)	Amplicon Size (bp)
***bla*_CTX-M G1_**	**G1 F** 5′-AAA AAT CAC TGC GCC AGT TC	415
	**G1 R** 5′-AGC TTA TTC ATC GCC ACG TT	
***bla*_CTX-M G2_**	**G2 F** 5′-CGACGCTAC CCCTGC TAT T	552
	**G2 R** 5′-CCAGCGTCAGAT TTT TCA GG	
***bla*_CTX-M G8_**	**G8 F** 5′-TCG CGT TAA GCG GAT GAT GC	666
	**G8/G25 R** 5′-AAC CCA CGA TGT GGG TAG C	
***bla*_CTX-M G9_**	**G9 F** 5′-CAA AGA GAG TGC AAC GGA TG	205
	**G9 R** 5′-ATT GGA AAG CGT TCA TCA CC	
***bla*_CTX-M G25_**	**G25 F** 5′-GCA CGA TGA CAT TCG GG	327
	**G8/G25 R** 5′-AAC CCA CGA TGT GGG TAG C	

**Table 3:** AmpC genes which were tested, nucleotide sequence (5′–3′) of sense (F) and antisense (R) primers used, and PCR product size for each primer (bp).

Genes	Primers Sequences (5′–3′)	Amplicon Size (bp)
***bla*** **_dha-1_** **, *bla*_dha-2_**	**dhaM F** 5′-ACA CTG ATT TCC GCT CTG CT-3′ **dhaM R** 5′-ACA ATC GCC ACC TGT TTT TC-3′	1043
***bla*** **_CMY_**	**CMY F** 5′-GGG CCC GGA CAC CYT TTT GC-3′ **CMY R** 5′-TAA GTG TAG ATG ACA RCA GG-3′	1256

**Table 4:** Compilation of phenotypic characterisation results of isolates from the various water samples, including the presumptive identification, the medium from which it was isolated, resistance mechanism, and the diameters of the zone of inhibition for each antibiotic.

					Antibiogram 1 (Inhibition Zone Diameter/mm)									Antibiogram 2 (Inhibition Zone Diameter/mm)								
WATER SAMPLE	ISOLATES	IDENTIFICATION	MEDIUM	RESISTANCE MECHANISM	AMP10	AMC30	CTX30	CAZ30	ATM30	FEP30	CPT30	FOX30	MEM10	CIP5	F300	TE30	FO200	TGC15	C30	SXT25	CN10	TOB10
**MAT1**	**MAT1**CTX1	Another	MacConkey + Cefotaxime	**AmpC +**	6	7	7	12	16	14	8	6	23	29	21	6	36	15	21	26	12	14
**Brito Capelo Street**	**MAT1**CTX2	*E. coli*	MacConkey + Cefotaxime	**AmpC +**	6	12	9	6	11	13	8	11	24	23	21	6	36	16	22	6	13	13
	**MAT1**CTX3	KESC	MacConkey + Cefotaxime	**ESBL −; AmpC +**	6	11	13	6	15	20	13	6	18	6	6	17	21	15	10	16	14	11
	**MAT1**MERO1	*Pseudomonas*	MacConkey + Meropenem	**Degradative (CIM +)**	6	6	21	19	17	24	22	6	6	34	6	19	6	15	20	26	18	17
**MAT2**	**MAT2**CTX8	*E. coli*	MacConkey + Cefotaxime	**AmpC +**	6	6	23	17	38	34	15	6	31	28	29	23	29	19	22	23	28	27
**Brito Capelo Street**	**MAT2**MERO3	*Pseudomonas*	MacConkey + Meropenem	**Non degradative (CIM −)**	6	6	15	19	19	21	12	6	25	30	6	6	11	6	6	6	25	20
	**MAT2**MERO5	KESC	MacConkey + Meropenem	**AmpC +; Non degradative (CIM −)**	6	6	20	11	22	27	19	6	24	6	10	17	24	20	15	17	26	12
	**MAT2**MERO7	*Pseudomonas*	MacConkey + Meropenem	**Non degradative (CIM −)**	17	19	24	23	21	26	22	14	32	28	12	21	18	24	11	24	25	23
	**MAT2**MERO9	*E. coli*	MacConkey + Meropenem	**ESBL +; Non degradative (CIM −)**	6	16	9	14	14	15	8	25	28	8	22	18	25	19	22	6	22	19
**MAT3**	**MAT3**CTX1	*E. coli*	MacConkey + Cefotaxime	**ESBL +**	6	17	20	28	34	29	13	26	33	25	30	22	18	25	23	24	20	20
**Riguinha Stream**	**MAT3**CTX2	*E. coli*	MacConkey + Cefotaxime	**ESBL +**	6	16	16	28	30	17	11	23	21	25	25	19	12	25	31	23	19	18
	**MAT3**CTX3	KESC	MacConkey + Cefotaxime	**ESBL +; Non degradative (CIM −)**	6	6	7	9	8	9	6	18	20	6	19	8	20	17	6	6	6	8
	**MAT3**CTX4	*E. coli*	MacConkey + Cefotaxime	**AmpC +**	6	6	20	15	20	25	19	6	20	25	24	19	34	22	21	26	19	19
	**MAT3**CTX5	*E. coli*	MacConkey + Cefotaxime	**ESBL +**	6	20	19	30	39	26	15	25	26	28	29	25	15	28	33	23	20	20
	**MAT3**MERO1	*E. coli*	MacConkey + Meropenem	**Degradative (CIM +); AmpC −**	13	6	32	24	36	33	27	19	19	28	27	22	28	23	21	21	22	20
	**MAT3**MERO2	*E. coli*	MacConkey + Meropenem	**Degradative (CIM +); AmpC −**	9	6	32	28	41	26	26	26	22	26	30	23	28	26	24	14	23	12
	**MAT3**MERO3	*E. coli*	MacConkey + Meropenem		6	6	21	20	36	23	17	24	17	22	27	15	30	19	21	6	24	12
	**MAT3**MERO4	*E. coli*	MacConkey + Meropenem	**Degradative (CIM +)**	15	6	12	30	27	29	23	14	18	23	24	19	24	25	18	22	21	18
	**MAT3**MERO5	*E. coli*	MacConkey + Meropenem	**Degradative (CIM +); ESBL −**	6	6	20	21	38	24	16	24	17	20	26	14	32	22	20	6	22	9
	**MAT3**MERO6	KESC	MacConkey + Meropenem	**Degradative (CIM +)**	6	6	31	15	32	30	16	12	12	24	26	15	28	23	21	25	17	14
**MAT4**	**MAT4**CTX1	KESC	MacConkey + Cefotaxime		6	14	10	13	15	13	10	22	28	13	21	7	28	21	24	19	20	21
**Riguinha Stream**	**MAT4**CTX2	*E. coli*	MacConkey + Cefotaxime	**ESBL +**	6	15	10	13	15	13	8	23	21	13	22	7	29	22	26	14	21	21
	**MAT4**MERO3	*E. coli*	MacConkey + Meropenem	**ESBL −**	6	11	34	29	31	31	22	26	24	23	25	13	25	20	20	6	21	23
	**MAT4**MERO4	*E. coli*	MacConkey + Meropenem	**ESBL +**	6	13	30	32	32	32	31	17	31	29	14	27	21	26	15	18	36	31
**MAT5**	**MAT5**CTX1	*E. coli*	MacConkey + Cefotaxime	**ESBL +**	6	8	6	14	15	16	6	20	30	6	22	15	28	21	20	6	12	14
**Riguinha Stream**	**MAT5**CTX2	KESC	MacConkey + Cefotaxime	**ESBL +**	6	6	6	9	11	10	6	21	25	10	18	14	18	18	24	6	11	9
	**MAT5**CTX3	*E. coli*	MacConkey + Cefotaxime	**AmpC +; Degradative (CIM +); ESBL −**	6	6	13	14	20	31	6	6	36	6	29	20	30	18	22	24	19	18
	**MAT5**CTX4	KESC	MacConkey + Cefotaxime	**ESBL +**	6	6	6	9	10	9	6	21	26	9	18	15	18	18	24	6	10	9
	**MAT5**MERO1	*E. coli*	MacConkey + Meropenem	**Degradative (CIM +)**	6	9	6	6	6	9	6	10	13	30	22	15	28	22	22	28	20	18
	**MAT5**MERO2	KESC	MacConkey + Meropenem		6	6	21	30	28	28	6	6	6	16	6	7	12	17	18	26	16	13
	**MAT5**MERO4	KESC	MacConkey + Meropenem	**Degradative (CIM +)**	6	9	11	15	26	27	6	6	17	17	16	12	23	18	14	22	22	12
**MAT6**	**MAT6**CTX1	KESC	MacConkey + Cefotaxime	**AmpC −**	6	7	6	14	38	27	7	6	22	24	27	24	26	21	28	27	20	16
**Brito Capelo Street**	**MAT6**CTX2	KESC	MacConkey + Cefotaxime	**Degradative (CIM +); ESBL −**	6	6	15	17	14	19	6	20	23	30	15	22	24	19	24	6	17	12
	**MAT6**CTX3	KESC	MacConkey + Cefotaxime	**AmpC +**	6	6	24	21	26	30	12	6	24	30	24	10	10	17	23	24	25	21
	**MAT6**CTX4	*E. coli*	MacConkey + Cefotaxime	**AmpC +**	6	6	6	10	15	26	6	6	22	21	18	11	20	18	20	26	20	18
	**MAT6**CTX5	*E. coli*	MacConkey + Cefotaxime	**ESBL +**	6	6	23	13	31	29	6	19	32	23	27	21	22	18	20	25	19	18
	**MAT6**MERO1 *	*Pseudomonas*	MacConkey + Meropenem	**Degradative (CIM +)**																		
	**MAT6**MERO2	KESC	MacConkey + Meropenem	**Degradative (CIM +)**	6	6	6	6	6	11	6	10	11	13	13	11	22	19	23	24	20	17

* For MAT6MERO1 isolate, disks for *Pseudomonas* spp., were considered adequate; therefore, the table does not show the corresponding inhibition for these antibiotics. Green cell—susceptible isolate; White cell—susceptible with increased exposure and, Red cell—resistant.

## Data Availability

All relevant data are included in this manuscript.
